# Microbial Diversity Under the Influence of Natural Gas Storage in a Deep Aquifer

**DOI:** 10.3389/fmicb.2021.688929

**Published:** 2021-10-13

**Authors:** Magali Ranchou-Peyruse, Marion Guignard, Franck Casteran, Maïder Abadie, Clémence Defois, Pierre Peyret, David Dequidt, Guilhem Caumette, Pierre Chiquet, Pierre Cézac, Anthony Ranchou-Peyruse

**Affiliations:** ^1^IPREM, Institut des Sciences Analytiques et de Physico-Chimie pour l’Environnement et les Matériaux, Université de Pau & Pays Adour/E2S-UPPA, Pau, France; ^2^Laboratoire de thermique, énergétique et procédés IPRA, EA1932, Université de Pau & Pays Adour/E2S-UPPA, Pau, France; ^3^Joint Laboratory SEnGA, UPPA-E2S-Teréga, Pau, France; ^4^Université Clermont Auvergne, INRAE, UMR 0454 MEDIS, Clermont-Ferrand, France; ^5^STORENGY – Geosciences Department, Bois-Colombes, France; ^6^Teréga, Pau, France

**Keywords:** deep aquifer, BTEX, biodegradation, deep subsurface biosphere, high pressure, benzyl-succinate synthase, gas storage

## Abstract

Deep aquifers (up to 2km deep) contain massive volumes of water harboring large and diverse microbial communities at high pressure. Aquifers are home to microbial ecosystems that participate in physicochemical balances. These microorganisms can positively or negatively interfere with subsurface (i) energy storage (CH_4_ and H_2_), (ii) CO_2_ sequestration; and (iii) resource (water, rare metals) exploitation. The aquifer studied here (720m deep, 37°C, 88bar) is naturally oligotrophic, with a total organic carbon content of <1mg.L^−1^ and a phosphate content of 0.02mg.L^−1^. The influence of natural gas storage locally generates different pressures and formation water displacements, but it also releases organic molecules such as monoaromatic hydrocarbons at the gas/water interface. The hydrocarbon biodegradation ability of the indigenous microbial community was evaluated in this work. The *in situ* microbial community was dominated by sulfate-reducing (e.g., Sva0485 lineage, Thermodesulfovibriona, *Desulfotomaculum*, *Desulfomonile*, and *Desulfovibrio*), fermentative (e.g., *Peptococcaceae* SCADC1_2_3, Anaerolineae lineage and *Pelotomaculum*), and homoacetogenic bacteria (“*Candidatus* Acetothermia”) with a few archaeal representatives (e.g., *Methanomassiliicoccaceae*, *Methanobacteriaceae*, and members of the Bathyarcheia class), suggesting a role of H_2_ in microenvironment functioning. Monoaromatic hydrocarbon biodegradation is carried out by sulfate reducers and favored by concentrated biomass and slightly acidic conditions, which suggests that biodegradation should preferably occur in biofilms present on the surfaces of aquifer rock, rather than by planktonic bacteria. A simplified bacterial community, which was able to degrade monoaromatic hydrocarbons at atmospheric pressure over several months, was selected for incubation experiments at *in situ* pressure (i.e., 90bar). These showed that the abundance of various bacterial genera was altered, while taxonomic diversity was mostly unchanged. The candidate phylum Acetothermia was characteristic of the community incubated at 90bar. This work suggests that even if pressures on the order of 90bar do not seem to select for obligate piezophilic organisms, modifications of the thermodynamic equilibria could favor different microbial assemblages from those observed at atmospheric pressure.

## Introduction

Among the different deep continental environments containing high total biomass levels, estimated at 2 to 6·10^29^ cells (Magnobosco et al., 2018), aquifers located in the uppermost two kilometers of the Earth’s crust are characterized by a diverse microbial biosphere (Wu et al., 2016; Hershey et al., 2018; Kadnikov et al., 2020). Oligotrophic conditions result in low cell concentrations; however, these aquifers have a considerable volume, estimated to be at 22.6 million km^3^ (Gleeson et al., 2015). Deep continental aquifers used as geological gas storage facilities are relevant ecosystems for studying the deep continental biosphere and the effect of human activity on it. These storage aquifers can be found all around the world, but are primarily located in North America, although some also occur in Europe and the Commonwealth of Independent States (Russia, Armenia, Azerbaijan, and Belarus; Cornot-Gandolphe, 2017). An understanding of these ecosystems makes it possible to predict the effects of future developments on these deep aquifers depending on their use, which include (i) the sequestration of CO_2_ or carbon molecules in the broad sense to address climate change; (ii) the temporary energy storage of CH_4_, H_2_ or air from renewable energy sources; (iii) rare metal mining; and (iv) exploitation of their water resources (De Silva et al., 2015; Flexer et al., 2018; Ranchou-Peyruse et al., 2019; Lemieux et al., 2020). Deep porous geological structures may present advantages such as their size, relative ubiquity and safety for gas storage, along with the proven know-how regarding their operation. Because of the high pressures deep underground, the volume of gas stored is less than that at atmospheric pressure, allowing the storage of large amounts of gas.

While the influence of high pressure on microbes has been widely studied in the context of marine environments (Jebbar et al., 2015; Zhang et al., 2015), very few such studies have been conducted on their continental counterparts (Escudero et al., 2018; Zhang et al., 2018). Nevertheless, international projects have aimed to fill the knowledge gaps in this theme, most notably including the following programs: the U.S Department of Energy’s Subsurface Program, Deep Underground Science and Engineering (DUSEL), the international Continental Scientific Drilling Program, the Deep Carbon Observatory’s Census of Deep Life, and the Center for Dark Energy Biosphere Investigations (C-DEBI). To our knowledge, no obligate piezophiles have yet been isolated from a deep continental environment, whereas they have been observed in marine subsurface environments. The temperature gradient of approximately 30°C km^−1^ implies that temperatures reached in deep continental environments prevent microbial life at depths of 3–4km. Multiple studies focusing on these environments were able to isolate and grow microorganisms at atmospheric pressure (Haveman and Pedersen, 2002; Baker et al., 2003; Basso et al., 2009; Leandro et al., 2018; Karnachuk et al., 2019). However, although the pressure encountered in these environments does not seem to select obligate piezophiles, pressure can influence the structure of the microbial community (Nyyssönen et al., 2014). In particular, pressure induces increased solubilization of gases and volatile carbon elements and could modify transfer rates through the cell membrane and accessibility to essential elements, including H_2_, CO_2_, and organic molecules (Follonier et al., 2012).

In the case of the geological storage of natural gas, deep aquifers are oligotrophic environments. The proximity of the stored gas has an impact on the microbial composition *via* the transfer of hydrocarbon molecules and other elements from the gas to water (Amigáň et al., 1990; Ranchou-Peyruse et al., 2019). Many of these environments contain sulfate, and heterotrophic sulfate-reducing microorganisms take advantage of this influx of organic molecules to prosper. In 2016, a study of the Pb_J_7 site in the Paris sedimentary basin closest to gas storage demonstrated the prevalence of sulfate-reducing microorganisms, particularly *Desulfotomaculum* spp., on the degradation of so-called BTEX monoaromatic hydrocarbons (Aüllo et al., 2016). These hydrocarbons are among the most soluble and thus the most likely ones to migrate in the aquifer. The study presented here concerns the Pb_J_6 site, which is part of the same Jurassic stratum as Pb_J_7 but is located at a distance of 1.3km. The main reason for this work was to test whether the monoaromatic hydrocarbon biodegradation potential was similar and involved the same microorganisms despite the aquifer being located farther away from the gas reservoir than the Pb_J_7 well. Moreover, with regard to deep aquifers used for natural gas storage, all biodegradation assays conducted to date have been carried out at atmospheric pressure, without taking into account the possible effects of *in situ* pressure on biodegradation capacities and microbial diversity. In this study, the Pb_J_6 monitoring well was cleaned using the same protocol used for sampling Pb_J_7 to prevent possible contamination by the biofilms developed in the pipes (Basso et al., 2005). The experiments carried out during this study combine classical microbiological approaches for assessing the BTEX biodegradation potential and molecular biology approaches for following changes of microbial diversity and understanding the behavior of these communities at atmospheric and *in situ* pressures.

## Materials and Methods

### Sample Site

The well is located approximately 20km west of Paris, and because it is a site of natural gas storage, it is classified as SEVESO-3.^1^ Its position with respect to other wells, particularly Pb_J_7, can be found in a publication by Ranchou-Peyruse et al. (2019). The well reaches a depth of 720 meters and has a temperature of 37°C. Depending on storage activity, the pressure can vary from 67 to 90bar. Formation water is contained in porous Jurassic rock primarily composed of sandstone, with some carbonates. Geochemical analyses of the waters were carried out by the CARSO Laboratory (France). The obtained data are presented in Table 1. The pH measured at the wellhead (i.e., surface) was 7.8. Samples were taken from the Pb_J_6 well located in the Paris sedimentary basin in September 2015 using the same aseptic and anoxic procedure described previously (Basso et al., 2005). Briefly, after brushing the entire well and circulating sodium hypochlorite (4.2L, 14% active chlorine) three times, the well was flushed (10 times the volume of the well, which is 2.4m^3^). A total of 99 Sterivex™-GP 0.22μm filter units (EMD Millipore, Molsheim, France) were used to filter the formation water and to concentrate the microbial biomass at a flow rate of 1.5m^3^ h^−1^ for 9h. The continuous flow of water made it possible to maintain anoxia inside the filters. At the end of the sampling campaign, the filters were sealed and immediately transported to the laboratory at anoxic conditions (GasPak™ EZ, BD) at room temperature. Several liters of additional formation water were also taken from the wellhead and stored in glass flasks at room temperature, respecting the conditions of anoxia during transport back to the laboratory. In the laboratory, the concentrated microbial biomass was stored at appropriate anoxic and sterile conditions in a glovebox at room temperature until use on the following day. Glass flasks with formation water were stored at 4°C until use, particularly for culture on the next day.

**Table 1:** Physicochemical parameters and components of the formation water from a deep aquifer analyzed at atmospheric pressure.

Temperature (°C)	37
Depth (m)	720
Pressure (bar)	88
pH	7.8
Conductivity (μS.cm^−1^)	2051
Eh (mV)	−284
Total phosphorus (mg.L^−1^ P)	<0.010
Total organic carbon, TOC (mg.L^−1^ C)	<1
Total suspended matter (mg.L^−1^)	3.8
**Gas analyses**	
H_2_S (mg.L^−1^)	0.70
Free CO_2_ (mg.L^−1^)	9.4
**Cations**	
NH_4_^+^ (mg.L^−1^)	2.5
Ca_2_^+^ (mg.L^−1^)	17.0
Mg_2_^+^ (mg.L^−1^)	17.01
Na^+^ (mg.L^−1^)	381.5
K^+^ (mg.L^−1^)	11.7
**Anions**	
CO_3_^2−^ (mg.L^−1^)	0
HCO_3_^−^ (mg.L^−1^)	628
Cl^−^ (mg.L^−1^)	178
SO_4_^2−^ (mg.L^−1^)	269
NO_3_^−^ (mg.L^−1^)	<0.1
NO_2_^−^ (mg.L^−1^)	<0.02
PO_4_^3−^ (mg.L^−1^)	0.02
F^−^ (mg.L^−1^)	10
Dissolved silicates (mg.L^−1^)	14.3
**Metals**	
Fe_total_ (mg.L^−1^)	<0.010
Fe^2+^ (mg.L^−1^)	0.09
Mn^2+^ (mg.L^−1^)	0.059
Mineralogy: sandstone with some carbonates	

### Culture Methods

#### Most Probable Number

In the laboratory, the formation water was used for cell counting *via* the most probable number (MPN) technique. One liter of the mineral base used to simulate formation water contained 0.0594g CaCl_2·_2H_2_O, 0.135g MgCl_2·_6H_2_O, 0.035g NaCl, 0.0152g SiO_2_, 0.02g KCl, 0.2g NH_4_Cl, 0.3g KH_2_PO_4_, 0.3g K_2_HPO_4_, 0.882g NaHCO_3_, 0.0013g FeCl_2·_4H_2_O, 0.5g cysteine as a reducing agent, 1mg resazurin as a redox indicator, and 10ml SL10 trace elements from a stock solution (Pfennig et al., 1981). Depending on the targeted metabolism (Table 2), the headspace of the basal medium was spiked with 1bar of H_2_/CO_2_ (80/20) for hydrogenotrophic methanogens; 5g of casein peptone, 1g of yeast extract, 6.8g of sodium acetate and 1.7g of NaNO_3_ for nitrate-reducing bacteria; or 15g of casein peptone, 10g of yeast extract and 2.5g of glucose for fermentative bacteria without the addition of a terminal electron acceptor (TEA). Following autoclaving (120°C, 20min), the various media were supplemented with 5ml of vitamins from a filtered and anoxic V10 stock solution (Pfennig et al., 1981). Finally, the pH was adjusted to 7.8. The media were then anoxically transferred into Hungate tubes. For MPN, 1ml of formation water was added to 9ml of medium. A series of successive dilutions were then performed until the final dilution of 10^−6^ (triplicate). All the cultures were incubated at 37°C. Growth was monitored daily by measuring turbidity (OD 600nm; spectrophotometer, Spectronic 401, Spectronic Instruments) and was finally confirmed by observation under a microscope (BX60, Olympus). The culture medium used to select sulfate reducers was a commercial medium (SRB Labège® Test kit, CFG, France). To count the sulfate-reducing spores present in the formation water, the water was heated to 80°C for 10min directly at the sampling site before being inoculated in the appropriate medium in the laboratory in accordance with the procedure described here.

**Table 2:** Results of metabolic group counts by MPN and BTEX degradation according to targeted metabolism.

Targeted metabolism in formation water	MPN^a^ (cells.ml^−1^)	Culture conditions for degradation assays				Degradation profiles (days)^c^					
Sulfate reducers Sporulated sulfate reducers (pH 7, 37°C)	2.5×10^1^ 0.9×10^1^	Consortia	TEA^b^ supplemented	pH	T°C	B	T	E	*m*-X	*o*-X	*p*-X
		A1	**SO**_**4**_^**2−**^	**7.2**	**37**	∞	49/117	249/333	49/117	∞	49/117
		Subcultured				∞	35/139	139/210	35/139	35/^*^	39/139
		A2	SO_4_^2−^	**6.55**	37	250/502	49/118	159/250	49/118	159/502	49/118
		Subcultured				210/373	35/139	35/373	35/139	35/373	35/139
		A3	SO_4_^2−^	**8.05**	37	∞	∞	∞	∞	∞	∞
		Not subcultured									
		A4	SO_4_^2−^	7.2	**42**	∞	49/122	∞	49/122	∞	49/122
		Subcultured				35/211	35/140	211/715	1/140	35/211	35/140
		A5	SO_4_^2−^	7.2	**60**	∞	∞	∞	∞	∞	∞
		Not subcultured									
Nitrate reducers	4.5×10^2^	B	**NO**_**3**_^**−**^	7.2	37	∞	∞	∞	∞	∞	∞
		Not subcultured									
No TEA	9.5×10^1^	C	**None**	7.2	37	∞	55/125	∞	55/125	∞	55/125
		Subcultured				∞	58/140	∞	1/58	∞	58/128
Hydrogenotrophic methanogens	0.9×10^1^	D (+H_2_)	**CO**_**2**_	7.2	37	∞	∞	∞	∞	∞	∞
		Not subcultured									

*B, benzene; T, toluene; E, ethylbenzene; o-X, ortho-xylene; p-X, para-xylene; m-X, meta-xylene; ∞, no degradation observed*.

* *after 210 days, degradation was still in progress but the assay was stopped*.

a *MPN, most probable number*.

b *TEA, terminal electron acceptor*.

c *Numbers indicate culture times at which degradation was detected/completed*.

#### Biodegradation Assays

Biodegradation assays of BTEXs were performed with site water supplemented with concentrated biomass, as described by Berlendis et al. (2010). A sample of this water enriched with microorganisms was stored (80ml) at −20°C for molecular biology analyses. Between the sample being taken and the initiation of the biodegradation assays (less than 24h), the water continued to change, primarily under the effect of degassing, resulting in a pH of 7.2. The biodegradation assays were performed with 4 different media, A, B, C and D, to reveal the metabolic groups implicated in BTEX biodegradation (Table 2). Different conditions were tested in microcosms A, favoring sulfate-reducing microorganisms. The preparation of the different microcosms was carried out as follows: the formation water of microcosms A1 (pH 7.2; 37°C), A2 (pH 6.55; 37°C), A3 (pH 8.05; 37°C), A4 (pH 7.2; 42°C), and A5 (pH 7.2; 60°C) was supplemented with 10mM Na_2_SO_4_; microcosm B was supplemented with 10mM NaNO_3_; no TEA was added to microcosm C (beyond the TEAs potentially present in the formation water); and an H_2_/CO_2_ (80/20; 1bar) gas phase was added to microcosm D. The biomass that was concentrated on the 99 filters (Sterivex, EMD, Millipore, Molsheim, France) during sampling was resuspended in 200ml of formation water in an anaerobic glovebox (Getinge La Calhene, France) before being used to inoculate the biodegradation assays (1:10, v/v). Finally, a mixture of benzene, toluene, ethylbenzene, and *m*-, *o*-, and *p*-xylenes (Sigma–Aldrich) was added to a final concentration of 10ppm (v/v). Each condition was tested in duplicate, and a third vial was used as an abiotic control following the addition of 0.5ml of 10N HCl. Subcultures were then prepared in formation water sterilized by filtration (0.2μm porosity, cellulose nitrate filters, Sartorius Stedim) and appropriately supplemented to adapt them to the target metabolism. BTEX was monitored using a 7890A gas chromatograph (Agilent Technologies) equipped with a flame ionization detector, employing the same equipment and the same procedure described by Aüllo et al. (2016).

#### Culture at 90Bar Pressure

At the end of the biodegradation experiment conducted at atmospheric pressure, the two A2 replicates (sulfate-reducing conditions) were pooled and used as a 10% (v/v) inoculum in the formation water previously sterilized by filtration at 0.2μm porosity. As above, this water was supplemented with SL10 trace elements, vitamins, dithionite (0.2% w/v) as a reducing agent, 9mM NH_4_Cl, 2mM K_2_HPO_4_, 2mM KH_2_PO_4_, 10mM Na_2_SO_4_, and BTEX (10ppm each). When the initiation of degradation was observed for the *p*- and *m*-xylenes, 360ml of the culture was transferred under sterile and anoxic conditions to a flask at atmospheric pressure and to a reactor in which the pressure was gradually increased up to 90bar, both at a temperature of 37°C. The experiment was performed with an apparatus consisting of a one-liter stainless steel reactor in which it was possible to implement reactions at up to 300bar and 200°C. This reactor had three potential feeders and one outlet line equipped with a gas–liquid separator (cyclonic to limit the drive of the liquid phase). A constant reaction temperature was maintained by the circulation of oil in an external jacket connected to a thermostat. A variable-speed stirrer agitated the mixture. Safety measures such as a rupture disc and high-temperature cutoff were also implemented. A complete description of the device is presented by Kriaa et al. (2009). The reactor was sterilized with distilled water at 120°C for 4h. Inside the reactor, BTEX levels were monitored using the same approach described above in the “Biodegradation assays” section. Sampling was conducted using a PEEK tube with a diameter of 0.22mm and length of 4m (calculated internal volume of 125μl) and heat sterilized (100°C, overnight). A slight decrease in pressure was recorded throughout the experiment due to the uncompensated sample volumes (bacterial cell count and BTEX quantification).

#### Cell Counts

Twenty-four hours after sampling, the proportion of live cells was estimated by epifluorescence microscopy using a LIVE/DEAD BacLight Bacterial viability kit (Thermo Fisher Scientific) from the formation water sampled to determine the possible impact of depressurization during ascent through the control well. For this purpose, 10ml of formation water was spiked with 25μl of the SYTO9/PI (v/v) mix. After 15min in darkness, the sample was filtered through 0.2μm pore-size black polycarbonate (25mm, Millipore) under vacuum. Moreover, the total cell concentrations in the formation water and concentrated biomass were estimated using epifluorescence microscopy with DAPI staining (4',6'-diamidino-2-phenylindole, Sigma-Aldrich). Eighteen milliliters of the water was fixed with 2ml of 10% borax-buffered formaldehyde (37%, Sigma-Aldrich) before being stored at 4°C until observation. Next, 10ml of the fixed sample was spiked with 900μl of DAPI stock solution (200μg.ml^−1^) before being filtered through 0.2μm pore-size black polycarbonate (25mm, Millipore) under vacuum. In the experiment at 90bar pressure, cell counting was conducted using 500μl of culture fixed with 100μl of a 10% borax-buffered formaldehyde stock solution and then spiked with 25μl of DAPI before being filtered through 0.2μm pore-size black polycarbonate (25mm, Millipore) under vacuum. In each of the measurements, 10 randomly selected fields were used for cell counting which represented 202 to 507 microbial cells counted. The Zeiss Observer Z1c epifluorescence microscope was equipped with a mercury light source.

### Molecular Biology Methods

#### Diversity of the Concentrated Biomass

Eighty milliliters of the water enriched with microorganisms used for the biodegradation tests was filtered (0.2μm porosity, cellulose nitrate filters, Sartorius Stedim). The filters were then ground in liquid nitrogen to a fine powder, and DNA was extracted using the Mobio-Power Soil DNA isolation kit (Mobio Laboratories, Carlsbad, CA) in line with the supplier’s recommendations. The genomic DNA was sent to a commercial company (MR DNA, Shallowater, Texas, United States). Following the amplification of the V4 hypervariable region of the 16S rRNA gene (PCR primers 515/806), the amplicons were sequenced *via* MiSeq 2 × 300bp sequencing (Illumina, California, United States) in line with the supplier’s recommendations. The sequences were converted and demultiplexed with QIIME1. The sequences were analyzed using the software package DADA2 (Callahan et al., 2016) in the QIIME2 pipeline. With this package, the sequences were treated by quality filtering, merging, dereplicating, and removing chimeras to determine amplicon sequence variants (ASVs). Taxonomy was assigned using VSearch (Rognes et al., 2016) against the SILVA SSU 138 NR database (Quast et al., 2013) without uncultured/environmental sequences. The raw data were deposited in the NCBI SRA under bioproject ID PRJNA739070.

#### Sequence Capture by Hybridization Experiment

The *bssA* gene encodes benzylsuccinate synthase, which initiates the anaerobic degradation of toluene and xylenes. A set of thirty-four 54- to 57-mer degenerate probes covering the *bssA* gene (Supplementary Table S1) was designed based on 17 *bssA* nucleic sequences from strains belonging to the phylum Desulfobacterota, which was recently defined from delta-Proteobacteria (EF123662, EF123663, EF123667, and EU780921), Firmicutes (EF123665, KJ398022, and GU357866), *Geobacteraceae* (AF123664, EF123666, and AF441130), and environmental sequences obtained with the 7772F/8546R primers set (KF995713, KF995719, KF995720, and KF995723). Three *bssA* sequences (KX576576, KX576577, and KX576575) were obtained from a previous study of deep aquifers (Ranchou-Peyruse et al., 2017). All of these sequences were processed using KASpOD software (Parisot et al., 2012), and we preferentially selected probes targeting the sequence encoding the Cys-loop (Funk et al., 2015). Adaptor sequences were added at each end of the probe to enable PCR amplification, resulting in the sequences “ATCGCACCAGCGTGT-N_(31–38)_-CACTGCGGCTCCTCA,” where N_(31–38)_ represents the *bssA*-specific capture probe. Biotinylated RNA capture probes were then synthesized as described by Ribière et al. (2016).

A next-generation sequencing library was prepared from the DNA extracted from microcosm B using the Nextera XT Kit (Illumina) following the manufacturer’s instructions. Solution hybrid selection (SHS) was conducted according to the protocol described by Ribière et al. (2016). Briefly, 500ng of the heat-denatured libraries was hybridized to the set of biotinylated RNA probes for 24h at 65°C. Probe/target heterodimers were trapped by streptavidin-coated paramagnetic beads (Dynabeads M-280 Steptavidin, Invitrogen, Carlsbag, CA, United States). After several washing steps, the captured targets were eluted from the beads using NaOH and purified using AMPure beads (Beckman Coulter Genomics, Takeley, Essex, United Kingdom). The enriched products were PCR amplified using primers complementary to the library adapters and purified again using AMPure beads (Beckman Coulter Genomics). To increase enrichment, a second round of hybridization and amplification was performed using the obtained captured products.

Captured DNA products were sequenced using a MiSeq 2×300bp run (Illumina) according to the manufacturer’s specifications (Genoscreen). All raw reads were scanned for library adaptors and quality filtered using PRINSEQ-lite PERL script (Schmieder and Edwards, 2011) prior to assembly and analysis. The clean reads were assembled *de novo* using IDBA-UD (v1.1.1; Peng et al., 2012). The contigs generated were combined for a second round of assembly using CAP3 to generate longer contigs (Huang and Madan, 1999). The amino acid (AA) sequences were deduced from the final assembled nucleotide contigs and then aligned with reference open-reading frames sourced from public databases using MEGA version 6 (Tamura et al., 2013). The two obtained *bssA* sequences were deposited in the NCBI database (MW762607 and MW762608).

#### Diversity of the Communities Cultivated at 90Bar Versus Atmospheric Pressure

After 120days of incubation, samples were taken from each of the two cultures kept at high pressure (220ml) and atmospheric pressure (335ml) and filtered through a 0.22μm pore-size filter. The filters were immediately cut into three equal parts under sterile conditions and stored at −80°C for RNA conservation until use. Each piece of the filter was then ground in liquid nitrogen, and the RNA was extracted using the FastRNA® Pro Soil-Indirect kit (MP Biomedicals, Inc.) and separated from the DNA using the AllPrep DNA/RNA FFPE kit (Qiagen) in line with the manufacturer’s recommendations. Although only 16S rRNA gene data were employed in this study, the whole meta-transcriptomic experiment was performed on 2 × 3 replicates by a commercial company (Sequentia Biotech, Barcelona, Spain). Total RNA was quantified on a QUBIT fluorometer. The libraries were produced using retrotranscribed cDNA previously amplified with an Ovation Ultralow Library System V2 (NuGEN Technologies, Inc.). Library size and integrity were assessed using an Agilent Bioanalyzer (Santa Clara, CA). Sequencing was performed on the Illumina HiSeq 2,500 (Illumina, San Diego, CA), and 30M paired-end reads (2×125) per sample were generated. Sequences that were too small (<35bp) or had low-quality scores (<25) were eliminated with BBDuk software (version 12/2015) to improve the analysis quality. The raw data were processed *via* SortmeRNA (Kopylova et al., 2012) through the ASaiM framework with Galaxy^2^ to select the 16S rRNA gene sequences from the R1 and R2 files of each triplicate set of samples (Batut et al., 2018). Next, the R1 and R2 sequences from each file were interlaced into a single file using FASTQ interlacer. The paired R1/R2 sequences were processed with DADA2 in the QIIME pipeline, as described earlier. The raw data were deposited in the NCBI SRA under bioproject ID PRJNA715357.

## Results

### Physical Chemistry of the Aquifer Water

The formation water taken from a depth of 720m (Table 1) evolved at a pressure of 88bar in a rock formation composed of sandstone and carbonates dates back to the Jurassic period (Malm Kimmeridgian-Oxfordian). The water is mesothermal (37°C) with a pH of 7.8. It is brackish water with a conductivity of 2051 μS.cm^−1^. This salinity is primarily controlled by four predominant ions: Na^+^ and HCO_3_^−^ (381.5 and 628mg.L^−1^, respectively), followed by SO_4_^2−^ (269mg.L^−1^) and Cl^−^ (178mg.L^−1^). The sum of these ions (1.456g.L^−1^) represents 4% of marine salinity. According to the Langelier–Ludwig diagram, HCO_3_^−^/Cl^−^>1, Na^+^/Cl^−^>1, and Na^+^/K^+^=32.6 ratios are characteristic of formation water under the influence of the rock composition. Free CO_2_ could be detected (9.4mg.L^−1^). With total organic carbon (TOC)<1mg.L^−1^ and Eh=−284mV, the conditions are oligotrophic and highly reducing. The presence of oxygen, nitrate or nitrite could not be demonstrated, but an H_2_S content of 0.70mg.L^−1^ was recorded. The ammonium concentration was relatively high, at 2.5mg.L^−1^, whereas the phosphate concentration was low, at 0.02mg.L^−1^.

### Composition of the Microbial Community From the Formation Water

**Figure 1:**
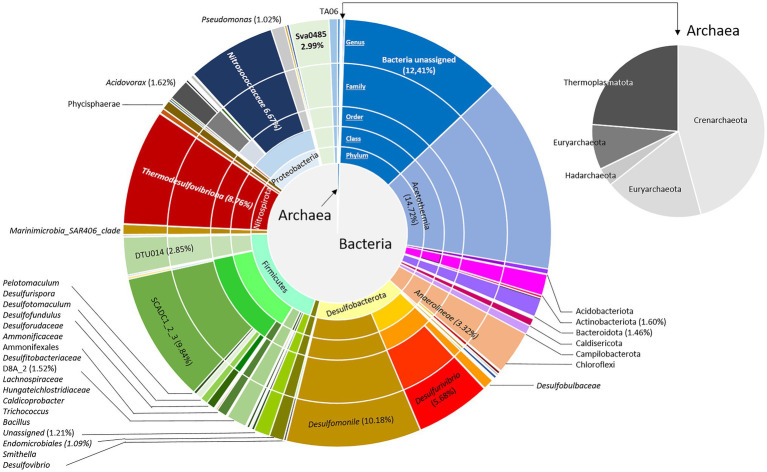
Bacterial and archaeal community composition in the formation water from a deep aquifer used for natural gas storage (Pb_J_6, Parisian sedimentary basin). Based on the V4 region of the 16S SSU rRNA, the pie charts represent the relative abundance as the percentage of each taxon with the interior toward the periphery: phylum, class, family, and genus.

Depending on the flow rate and the volume of the well, the rise of a microbial cell from the aquifer to the surface is estimated to last for approximately 100min. The impact of decompression from 88bar to atmospheric pressure during water wellhead sampling appears to be relatively low given the microorganism survival rate of 80% estimated using the LIVE/DEAD microscopy technique 24h after obtaining the sample. The cell number present in the water was 2.43·10^4^ cells.ml^−1^. This biomass was concentrated approximately 25 times by the addition of biomass from Sterivex filters to reach a concentration of 6.10·10^5^ cells.ml^−1^. Taxonomic diversity was then assessed based on the sequencing and analysis of 16S rRNA genes from the microbial community, and 10,741 amplicon sequence variants (ASVs) were generated (Figure 1). In total, 23 bacterial and 5 archaeal phyla were revealed in the concentrated biomass from the formation water. The microbial community was largely dominated by bacteria, which showed a relative abundance of 99.4% of ASVs. Excluding unassigned bacteria (12.4%), the microbial community was mainly composed of 10 different phyla: Firmicutes (19.7%), Desulfobacterota (17.4%), “*Candidatus* Acetothermia” (14.7%), Proteobacteria (10.4%), Nitrospirota (8.8%), Chloroflexi (3.3%), Sv0485 (3.0%), Actinobacteriota (1.6%), Bacteroidota (1.5%), and Elusimicrobiota (1.1%). The microbial community was driven by fermenters and sulfate reducers such as “*Candidatus* Acetothermia,” *Peptococcaceae* SCADC1_2_3, Anaerolineae, Desulfobacterota, Thermodesulfovibriona, and the candidate Sva0485 clade of the Deltaproteobacteria. Among the Proteobacteria, the microorganisms were mainly Nitrosococcaceae (6.7%), *Acidovorax* (1.6%), and *Pseudomonas* (1.0%).

The Archaean community was divided among Crenarchaeota (45.7%; represented by the Class Bathyarchaeia), Thermoplasmatota (23.7%; represented by the family *Methanomassiliicoccaceae*), Euryarchaeota (18.6%; represented by the family *Methanobacteriaceae*), Halobacterota (5%; represented by the family *Methanosarcinaceae*) and Hadarchaeota (3.4%; represented by the order Methanobacteriales).

When natural gas is stored in a deep aquifer, some of the BTEX contained in the gas can dissolve in the formation water and be degraded by microorganisms if these organisms have the potential to do so. Thus, the enrichment of this microbial community under several different conditions (different TEAs, temperatures or pH levels) was subsequently carried out.

### BTEX Enrichment Cultures at Atmospheric Pressure

The results of metabolic group enumeration in the on-site samples and the BTEX degradation profiles under various culture conditions are shown in Table 2. Counting using the MPN technique revealed a sulfate reducer concentration of 2.5·10^1^ cells.ml^−1^. On site, the samples were boiled for 10min to inactivate cells and evaluate the relative proportion of spores of sulfate-reducing bacteria, which showed a concentration of 0.9·10^1^ cells.ml^−1^. Microorganisms capable of fermentation and those capable of lithoautotrophic growth based on hydrogen showed concentrations of 9.5·10^1^ and 0.9·10^1^ cells.ml^−1^, respectively. Even though no nitrate was detected in the formation water, the proportion of nitrate-reducing microorganisms was relatively high, with a concentration of 4.5·10^2^ cells.ml^−1^. There was no growth in the presence of FeIII or oxygen in media targeting *Pseudomonas*, oligotrophs, methylotrophs and heterotrophs.

When selective enrichment conditions for the four metabolic groups for which growth had been demonstrated using the MPN technique were maintained, only sulfate-reducing (Conditions A) and fermentation (Condition C) conditions showed the capacity to degrade monoaromatic hydrocarbons. Without any TEA (fermentation, Condition C), only toluene, *m*- and *p*-xylene could be degraded. These compounds disappeared completely after approximately 4months. No degradation of benzene, ethylbenzene or *o*-xylene was observed.

Under sulfate-reducing conditions, several pH values and temperatures were tested. Degradation took place at 37 and 42°C but not at 60°C. While degradation was demonstrated at pH 7.2 and 6.55, no degradation was observed at pH 8.05. It should be noted that the aromatic hydrocarbons that were degraded first (toluene, *m*-xylene, and *p*-xylene) were the same than those that degraded under fermentation conditions. In addition to these three molecules, ethyl-benzene, *o*-xylene, and benzene were biodegraded under sulfate-reducing conditions over longer periods exceeding 1year.

While the anaerobic biodegradation capacity of BTEX has been the subject of numerous studies, the difficulty of accessing deep aquifers has caused these environments to be much less studied in general, particularly with regard to this type of metabolism. It is therefore important to detect the genes involved in this biodegradation process to reveal their diversity to better understand the phenomena *in situ*.

### bssA and bssA-Like Sequence Captured by Hybridization

**Figure 2:**
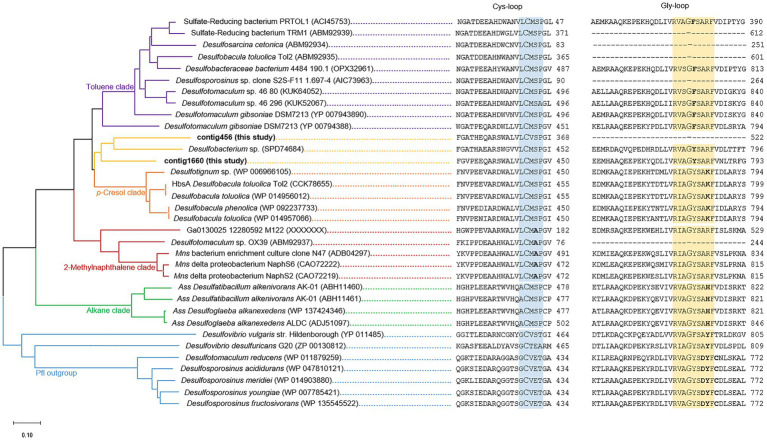
Phylogenetic tree based on the partial bssA-like amino acid sequences from a BTEX biodegradation assay (**in bold**; this study) compared with the fumarate-adding enzyme (FAE) sequences available in international databases. The evolutionary history was inferred using the neighbor-joining method (Saitou and Nei, 1987). The optimal tree with the sum of branch length=6.67348502 is shown. The tree is drawn to scale, with branch lengths in the same units as the evolutionary distances used to infer the phylogenetic tree. The evolutionary distances were computed using the Poisson correction method (Zuckerkandl and Pauling, 1965) and are presented in units of the number of amino acid substitutions per site. This analysis involved 34 amino acid sequences. All ambiguous positions were removed from each sequence pair (pairwise deletion option). There were a total of 914 positions in the final dataset. Evolutionary analyses were conducted in MEGA X (Kumar et al., 2018).

The enzyme responsible for the anaerobic biodegradation of hydrocarbons such as toluene and xylene isomers is benzylsuccinate synthase, which is encoded by the *bssA* gene. This gene was targeted in a biodegradation assay selecting for sulfate-reducing conditions (A1: pH 7.2, 37°C, Table 2), in which all the TEXs were degraded after 210days of incubation. Two amino acid sequences (contig456 with 522 AA and contig1660 with 807 AA) were related to bssA sequences. Figure 2 presents a phylogenetic tree constructed from fumarate-adding enzyme (FAE) amino acid sequences similar to enzymes reported to degrade hydrocarbons, particularly aromatics (2-methylnaphthalene, *p*-cresol, and toluene). Contig456 and contig1660 were in the same cluster as *Desulfobacterium* sp. (SPD74684), with 58% identity. This cluster was located between the bssA clades of *p*-cresol and toluene. While the Cys-loop, which plays a role in fumarate attachment, was clearly observed, the gly-loop contained the tyrosine (Y) amino acid characteristic of the *p*-cresol clade but the arginine (R) amino acid characteristic of the toluene clade.

### Culture at 90Bar vs. Culturing at Atmospheric Pressure

**Figure 3:**
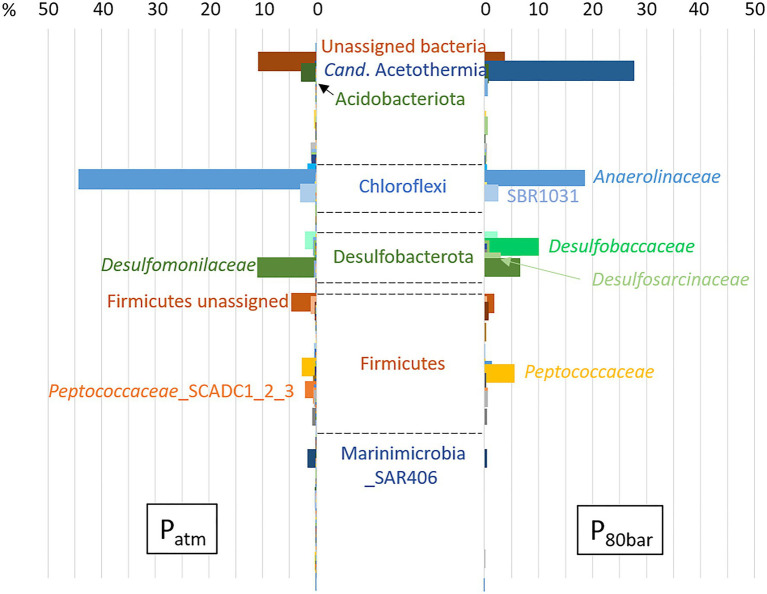
Degradation of BTEX by the bacterial community at 90 bar with monitoring of the cell concentration by microscopic counting.

**Figure 4:**
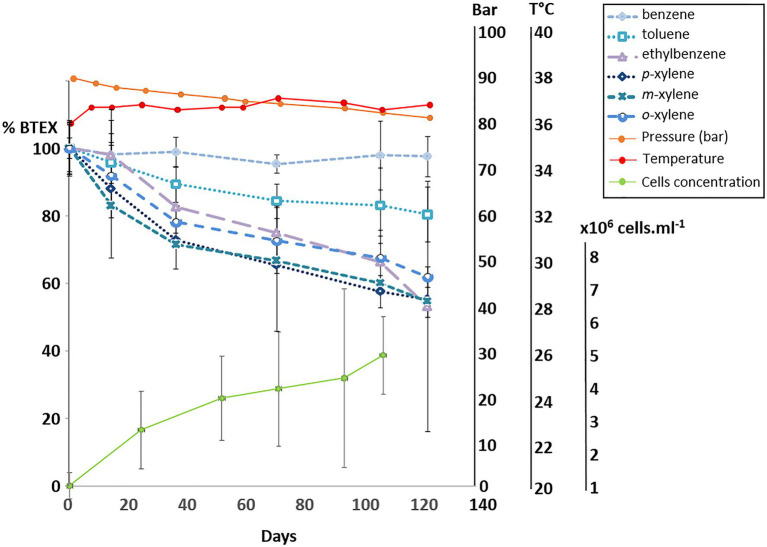
Relative abundance of active BTEX-degrading bacterial populations from a deep aquifer with or without pressure (90 bar). The percentage is shown on the X-axis. Total RNA was extracted from both communities, and taxonomic diversity analysis was performed based on 16S rRNA transcripts. The archaeal fraction accounted for less than 0.03% of ASVs.

Using the same biodegradation assay employed for the gene capture technique previously described, incubation at 90bar pressure and in the presence of the 6 aromatic hydrocarbon molecules was performed for a period of 120days (Figure 3). Throughout this period, slow cell growth was observed from 1.15·10^6^ to 5.11·10^6^ cells.ml^−1^ after 105days of incubation, in parallel with the disappearance of monoaromatic hydrocarbons (between 20 and 40%) with the exception of benzene. The taxonomic diversity of the active microbial community was analyzed at the two different pressure conditions at the end of incubation following RNA extraction. This community capable of degrading BTEX was largely dominated by bacteria (99.97% of ASVs), and the archaeal component of the initial community (Figure 1) was considerably reduced during the two successive incubations of consortium A1 and its subculture under sulfate-reducing conditions (Table 2) preceding incubation at 90bar pressure. At atmospheric pressure, 10.8% of the ASVs could not be assigned taxonomically when they represented only 3.4% of the at high pressure. Among the 49 bacterial phyla identified in the community, the 6 phyla highlighted in Figure 4 represented 83.6 and 90.9% of the taxonomic diversity at P_atm_ and P_90bar_, respectively. The phylum “*Candidatus* Acetothermia,” which was almost absent at atmospheric pressure, was highly overrepresented at high pressure (P_atm_: 0.3% vs. P_90bar_: 27.8%). The phylum Acidobacteriota was predominantly represented by the class Aminicenantia (P_atm_: 2.8% vs. P_90bar_: 0.8%). The Chloroflexi phylum was dominated by bacteria belonging to the Anaerolineae lineage in the *Anaerolinaceae* family, which represented the major microorganisms at atmospheric pressure, accounting for 44.1% of ASVs versus 18.7% at 90bar, as well as the SBR1031 lineage. The latter lineage was stable between the two culture conditions (2.9 vs. 2.5%). In the presence of 10mM sulfate, sulfate reducers were highlighted by the results as expected, particularly the phylum Desulfobacterota, which represented 14.9 and 24.9% of ASVs at P_atm_ and P_90bar_, respectively. The *Desulfobaccaceae* (0.6 vs. 10.2%) and *Desulfosarcinaceae* (0.3 vs. 4.2%) families were favored by pressure, while to a lesser extent, the members of the *Desulfomonilaceae* family seemed to benefit from conditions involving atmospheric pressure (10.9 vs. 6.6%). Regarding the phylum Firmicutes, a not negligible proportion of the ASVs remained unassigned (4.6 vs. 1.9%). This phylum was dominated by the *Peptococcaceae* family, comprising the lineage SCADC1_2_3 (2.1 vs. 0.1%), but also the genera *Desulfofarcimen* (formerly *Desulfotomaculum*; 0.0 vs. 5.6%) and *Cryptanaerobacter* (2.7 vs. 0.2%). The last major phylum was the candidate phylum Marinimicrobia_SAR406 (1.5 vs. 0.5%). At least 13.2% at atmospheric pressure and 21.5% at 90 bar of the ASVs were unassigned or assigned to candidate lineages.

## Discussion

Approximately 85 natural gas storage sites in aquifers can be found around the world (Cornot-Gandolphe, 2017). The current investigation focused on water from the Pb_J_6 site, which forms part of a group of sites studied to examine the diversity of sulfate reducers and methanogens in 2019 by Ranchou-Peyruse and collaborators. This previous work showed the importance of the genus *Desulfotomaculum* within the family *Peptococcaceae* in the phylum Firmicutes as well as archaeal members of the family *Methanosarcinaceae*. The Pb_J_6 site is located 1.3km from another site (Pb_J_7; Ranchou-Peyruse et al., 2019) and has also been studied to evaluate the BTEX biodegradation capacity of its microbial community (Aüllo et al., 2016). Although the two sites belong to the same geological gas storage structure, their distance is relatively significant given the estimated natural displacement of the water mass of 1m/year, or possibly less (Jost et al., 2007). As shown in Table 1, the only terminal electron acceptors (TEAs) present in these ecosystems in sufficient quantities to have a significant influence driving the functioning of these communities were sulfate (269mg.L^−1^) and CO_2_ (9.4mg.L^−1^)/bicarbonate (628mg.L^−1^). A large part of the community is represented by microorganisms related to sulfate reducers: *Desulfobulbaceae*, *Desulfomonile*, *Desulfovibrio*, *Desulforudaceae*, *Desulfofundulus*, *Desulfotomaculum*, and *Thermodesulfovibrio*. Moreover, the candidate Sva0485 clade is assumed to be a potential sulfate reducer (Tan et al., 2019).

### Taxonomic Diversity of the Microbial Community Found in Formation Water

During sampling and the 720-meter ascent to the surface, the dissolved gases escaped rapidly from the water and microorganisms. This degassing phenomenon was accentuated near stored natural gas and may lead to the deterioration of many cell membranes. By carrying out the ascent in approximately 100min, the objective was to limit microbial lysis, and the result was satisfactory, since 80% of the cells presented an intact membrane 24h after sampling. Almost half of the cultivable sulfate reducers (0.9·10^1^ cells.ml^−1^) could be related to *Peptococcaceae* because of their capacity to sporulate and develop in sulfate-reducing conditions (Table 2). The presence of the microorganisms identified during this study is consistent with other studies relating to various deep continental environments, in particular deep aquifers (Basso et al., 2009; Mu et al., 2014; Frank et al., 2016; Kadnikov et al., 2017; Gulliver et al., 2019; Karnachuk et al., 2019; Soares et al., 2019; Stemple et al., 2021). However, the discovery of several taxa of bacteria associated with aerobic or nitrate-reducing metabolism identified here does not seem to be compatible with what we currently know about the physicochemical conditions of deep aquifers. While numerous studies have alluded to microorganisms described as being strictly aerobic or as nitrate-reducers in deep continental environments, their presence remains controversial (Pedersen et al., 2008; Puente-Sánchez et al., 2018; Kadnikov et al., 2020). Several examples of oxygen detection are described in the literature, even though this kind of environment is expected to be deprived of it (Frank et al., 2016; Kadnikov et al., 2018). In this study, no growth was revealed under oxic conditions. Conversely, nitrate-supplemented MPN presented higher concentrations of microorganisms (4.5·10^2^ cells.ml^−1^) than MPN supplemented with other TEAs or without TEAs (fermentation), which were all below or equal to 9.5·10^1^ cells.ml^−1^. The dissolution of nitrate salts in some rocks, such as siltstone and sandstone, is theoretically possible and was suggested as a possible explanation for the presence of nitrate in some groundwater from eastern Utah and other aquifers (Stewart and Peterson, 1914; Holloway and Dahlgren, 2002). In natural gas storage aquifers, the successive injection and withdrawal of natural gas could (i) increase the movement of the water mass; (ii) produce pressure variations inducing the displacement of mineral particles (i.e., geomechanics); and (iii) induce the dissolution of trapped nitrate salts consumed by nitrate reducers (*Pseudomonas*, *Acidovorax*, and *Desulfurivibrio*). On the other hand, microorganisms that are assumed to use oxygen, nitrate or nitrite as TEAs may be maintained in these environments *via* the use of other TEAs yet to be defined or by fermenting the rare organic molecules present. For example, the genus *Pseudomonas* has been suggested to carry out dissimilatory sulfate reduction (Kliushnikova et al., 1992), and some members, such as *P. aeruginosa*, have shown the capacity to ferment amino acids (arginine) or pyruvate (Vander Wauven et al., 1984; Schreiber et al., 2006).

The studied environment is naturally oligotrophic (TOC<1mg.L^−1^ C), and the only sources of organic carbon available for microorganisms are probably recalcitrant organic matter sequestered in the rock pore space following sedimentation as well as hydrocarbons from the stored gas (Parkes et al., 2000; Ranchou-Peyruse et al., 2019). Several of the microorganisms identified in the community (Figures 1, 3) have representatives that have been found in hydrocarbon-rich deep subsurface environments. For example, the lineage *Peptococcaceae* SCADC1_2_3, the genus *Thermodesulfovibrio* and the phylum “*Ca*. Acetothermia” have been identified in oil fields (Nunn, 2015; Hu et al., 2016; Liang et al., 2016). The genera *Pelotomaculum*, *Desulfotomaculum, Desulfovibrio*, *Desulfomonile*, *Acidovorax*, and *Methanobacterium* have been detected in contaminated aquifers and/or coal-bearing sediments (Ulrich and Edwards, 2003; Penner et al., 2010; Gründger et al., 2015; Aüllo et al., 2016; Ranchou-Peyruse et al., 2019). The sulfate-reducing bacterial group Sva0485 and the genus *Thermodesulfovibrio* have been shown to be responsible for the degradation of monoaromatic hydrocarbons such as benzene and alkanes, which is consistent with our observations (Ulrich and Edwards, 2003; Liang et al., 2016).

Deep subsurface environments have been shown to favor the sporulating sulfate-reducing and fermentative microorganisms of the Firmicutes phylum (nearly 20% of the observed community here), which were represented at the Pb_J_6 site by *Peptococcaceae* SCADC1_2_3 and the genera *Pelotomaculum* and *Desulfotomaculum* in particular (Aüllo et al., 2013). Beyond the phylum Firmicutes, sulfate reducers and fermenters dominated the community, as the taxa “Ca. Acetothermia,” Desulfobacterota, and Nitrospirota and the Sva0485 lineage were abundant. In addition, fermentative microorganisms may also have the capability to produce hydrogen. In such energy-poor environments, hydrogen is assumed to be rapidly consumed by other community members, such as hydrogenotrophic methanogens (with less than 0.55% ASVs), syntrophic acetate-oxidizing bacteria (Firmicutes DTU014; Dyksma et al., 2020), homoacetogens (“Ca. Acetothermia”; Youssef et al., 2019), or hydrogenotrophic sulfate-reducing bacteria (*Desulfomonile*, *Desulfovibrio*, and *Desulfotomaculum*; DeWeerd et al., 1991; Voordouw, 1995; Aüllo et al., 2013). Although the Pb_J_6 site is located farther from the stored gas than the Pb_J_7 site, the presence of stored gas may still be a key factor in the structuring of the community, particularly since it is a source of organic molecules. Sulfate reducers and fermenters may lead to the degradation of organic compounds ranging from short-chain alkanes to more complex polymers *via* monoaromatic hydrocarbons (Table 2) and other polyaromatic compounds.

### Monoaromatic Hydrocarbon Degradation

Sulfate reducers (e.g., *Desulfotomaculum* and *Desulfovibrio*) and fermenters (e.g., *Pelotomaculum*) are known to belong to the key metabolic groups involved in the degradation of BTEX in deep aquifers (Berlendis et al., 2010; Kleinsteuber et al., 2012; Aüllo et al., 2016; Ranchou-Peyruse et al., 2019). In this study, BTEX compounds were used to model the transfer of organic molecules from the geologically stored gas phase to the aquifer. BTEX compounds were selected because these compounds, particularly benzene, are the most soluble hydrocarbons. In addition to fermentative microorganisms (degrading toluene, *m*-xylene, and *p*-xylene; Table 2), sulfate-reducing microorganisms may also degrade benzene, ethylbenzene and *o*-xylene if environmental factors are favorable. The Sva085 taxon consists of sulfate reducers, which have been shown to be capable of degrading benzene (Ulrich and Edwards, 2003). This capacity is consistent with the existence of these microorganisms in an oligotrophic environment near stored natural gas, in which hydrocarbons, particularly benzene, may be present and serve as nutrient resources. When BTEX compounds represent the only sources of carbon and energy accessible to this community, the microbial community undergoes simplification, with the disappearance of almost the entire archaeal fraction in particular (<0.03% of ASVs; Figure 4). The simplified microbial community remained capable of degrading all of the BTEX compounds but did so sequentially. The most rapidly degraded molecules were toluene, *m*- and *p*-xylene, with very similar degradation times. Once these three molecules disappeared, ethylbenzene and *o*-xylene were degraded. In the past, it has been demonstrated that while the presence of toluene does not interfere with the degradation of *m*-xylene (and inversely), the presence of one of these molecules delays the degradation of *o*- and *p*-xylene (Meckenstock et al., 2004; Morasch et al., 2004). Under certain pH and temperature conditions, benzene may finally be degraded by the microbial community. The positive effect of a pH of 6.5 on benzene degradation has already been demonstrated in a microbial community from another deep aquifer (Berlendis et al., 2010). Previous work suggested that biofilm growth should be largely dominant in deep subsurface environments, thus allowing microorganisms to benefit from more minerals by being in close contact to them (Wu et al., 2017; Casar et al., 2021). Based on these suggestions, we deduce that the degradation of BTEX compounds, particularly benzene, in deep aquifers should preferentially take place in biofilms at the rock–water interface, which less involvement of planktonic microorganisms in formation water at higher pH levels. It has been established that microorganisms can influence the pH in their immediate environment (Liermann et al., 2000). For example, research conducted on *Desulfovibrio fructosovorans* has shown that in medium with a pH of 8.5, this microorganism releases acid, thereby maintaining a pH of 6.7 in the close vicinity of its cells (Daumas et al., 1993).

The enzyme involved in anaerobic TEX degradation is benzylsuccinate synthase. Interestingly, previous studies of isolated bacterial strains capable of TEX degradation suggest that this enzyme can degrade toluene (TRM1 and TOL2 strains) and sometimes other alkylbenzenes (e.g., *o*- and *p*-xylene for the PRTOL1 strain; Rabus and Widdel, 1995; Heider et al., 2016). The metagenomic method applied in this study to evaluate the diversity of *bssA*-carrying organisms was shown to be very stringent and even made it possible to reveal rare diversity (Ranchou-Peyruse et al., 2017). Although the frequent degradation of methyl-substituted aromatic compounds was observed in this study, only two different, phylogenetically similar, benzylsuccinate synthase genes were identified within the low-diversity bacterial community. The two identified *bssA* genes were found to group closely with a *bssA* gene (SPD74684) affiliated with an uncultured *Desulfobacterium* species from a natural deposit of asphalt. They clustered between the BssA clade related to the degradation of toluene or other alkylbenzenes and the BssA clade related to *p*-cresol degradation. In the latter group, the model microorganisms *Desulfobacula toluolica* and *D*. *phenolica* are both capable of degrading toluene and *p*-cresol, while only *D. phenolica* can degrade phenol (Rabus and Widdel, 1995; Kuever et al., 2001). *D*. *toluolica* has also been shown to be capable of degrading *o*- and *p*-xylene but incapable of degrading *m*-xylene (Beller et al., 1996). It has been further demonstrated that this microorganism possesses an operon enabling the degradation of *p*-cresol and another operon enabling the degradation of toluene (Wöhlbrand et al., 2012). Like all glycyl radical enzymes, the two new BssA sequences described here possess a well-conserved Cys-loop. It should be noted that thse amino acid “methionine” directly following the “cysteine” active site is replaced by a “valine” in the case of contig456. The Gly-loop presents a “tyrosine” immediately after the “glycine” site, as do the BssA sequences of the clades of *p*-cresol, methylnaphthalene and alkanes. The ability to degrade aromatic compounds may represent a competitive advantage. Under pristine conditions, microbial degraders could use recalcitrant organic molecules trapped in rocks. Under natural gas storage conditions, a few ppm or even ppb of monoaromatic hydrocarbons available from the gas could feed these microorganisms.

### Cultivation at High Pressure

In the context of BTEX degradation in deep subsurface environments, no study simulating *in situ* pressure conditions has ever been conducted to our knowledge. Here, the selected bacterial community capable of BTEX degradation was cultivated at a pressure of 90bar (Figure 3). Throughout the 120-day incubation period, alkylbenzene degradation was demonstrated, along with slow bacterial cell growth characteristic of deep oligotrophic ecosystems (Chapelle and Lovley, 1990; Hoehler and Jørgensen, 2013). The incubation time may have been insufficient to observe benzene degradation at the applied pressure conditions, since the degradation of benzene in the biodegradation assays began after 200days. Samples of the liquid phase necessary to monitor BTEX and microorganism concentrations led to a slow decrease in pressure, and the incubation stopped when the pressure was 80bar. At atmospheric and high-pressure conditions, the relative abundances of the active microbial populations presented in Figure 4 highlight the dominance of *Bacteria*. Independent of the pressure condition, the community was dominated by fermenters and sulfate reducers. These fermenters, which produce acetate and hydrogen, belonged to the phylum Chloroflexi, specifically to the Anaerolinae lineage, *Anaerolinaceae* family and SBR101 group, and to the phylum Firmicutes and family *Peptococcaceae,* comprising specific members of the genus *Cryptanaerobacter* and the group SCADC1_2_3 (Kunapuli et al., 2007; Tan et al., 2014; Liang et al., 2015; Xia et al., 2016). Sulfate reducers were mainly grouped together in the phylum Desulfobacterota, with the families *Desulfobaccaceae*, *Desulfomonilaceae*, and *Desulfosarcinaceae*, but also in the phylum Firmicutes, with the genus *Desulfofarcimen* (family *Peptococcaceae*), and the phylum Acidobacteriota (Hausmann et al., 2018). Members of *Desulfobaccaceae* and *Desulfosarcinaceae* have been reported to use acetate as the sole source of carbon and electron donors (Galushko and Kuever, 2019, Watanabe et al., 2020). Likewise, the genus *Desulfofarcimen,* formerly *Desulfotomaculum*, includes the type species *D. acetoxidans* and *D. intricatum,* both of which are capable of oxidizing acetate (Spring et al., 2009; Watanabe et al., 2013, 2018). Most of these sulfate-reducers or fermenting microorganisms have already been shown to be involved directly or indirectly in the degradation of aliphatic and/or aromatic hydrocarbons (Kunapuli et al., 2007; Tischer et al., 2013; Tan et al., 2014; Liang et al., 2015; Watanabe et al., 2017). In particular, the genus *Cryptanaerobacter*, formerly *Desulfotomaculum* subcluster Ih, has already been shown to be involved in the degradation of monoaromatic hydrocarbons *via* isotopic approaches (Kunapuli et al., 2007; Kleinsteuber et al., 2008; Herrmann et al., 2010; Taubert et al., 2012). The degradation of these compounds *via* fermentation pathways leads to the production of acetate, CO_2_ and H_2_ in particular. It is therefore probable that monoaromatic hydrocarbons could have fueled acetotrophic and/or hydrogenotrophic bacteria. Here, the candidate phylum Acetothermia, described as a producer of acetate from CO_2_ and H_2_ or *via* fermentation (Hao et al., 2018; Youssef et al., 2019), was characteristic of the community incubated at 90bar. While this phylum was not existent at atmospheric pressure (0.3%), its relative representativeness rose to 27.8% at high pressure. This lineage has been described in several oligotrophic thermal environments, such as hot springs, deep-sea sediments or deep aquifers (Takami et al., 2012; Badhai et al., 2015; Kadnikov et al., 2017; Zaitseva et al., 2017; Varliero et al., 2019). Although it is not possible to determine how incubation at 90bar could have favored the growth of these microorganisms, it is obvious that this condition favored its activity and is a parameter that must be considered to isolate bacteria of this candidate group. The majority of studies that have detected the presence of this lineage focused on taxonomic diversity analyses carried out directly on environmental samples. Nevertheless, a study carried out on anaerobic digesters, which contain a complex community, also identified these microorganisms (Hao et al., 2018). We show that these microorganisms can be maintained and active in a simplified community cultivated at oligotrophic conditions and high pressure. These elements should be taken into account in future procedures to isolate the first representative(s) from this candidate group.

## Conclusion

The results showed that even at sites located away from stored gas, the microbial communities in deep aquifers retained the ability to degrade the 6 monoaromatic compounds at sulfate and/or fermentation conditions.

The experiment simulating the *in situ* pressure confirmed the maintenance of the biodegradation capacity when BTEX was the only source of carbon and electrons. On the other hand, the results showed that the pressure could influence the structure of the microbial community. Even at pressures below 100bar, the application of pressure seems to allow the culture of microorganisms that are not necessarily obligate piezophiles but have thus far been identified only through the observation of candidate lineages.

Finally, all the indicators led to the hypothesis that the driving force behind BTEX degradation in these environments should be biofilms. It is therefore necessary to study these processes in biofilm conditions and, hence, take into consideration a mineral phase colonized by microorganisms.

## Data Availability Statement

The datasets presented in this study can be found in online repositories. The names of the repository/repositories and accession number(s) can be found at: https://www.ncbi.nlm.nih.gov/, from MW787045 to MW788027; MW762607, MW762608; PRJNA715357.

## Author Contributions

MR-P, MG, FC, PCe, and AR-P co-conceived the study. MR-P, MG, MA, and AR-P performed the culture and molecular biology experiments. CD and PP carried out the DNA hybridization experiments. All authors contributed to the interpretation of the results and paper writing. All authors contributed to the article and approved the submitted version.

## Funding

Storengy and Teréga funded this research project. MR-P’s salary was supported by E2S-UPPA.

## Conflict of Interest

DD is employed by Storengy. GC and PCh are employed by Teréga.

The remaining authors declare that the research was conducted in the absence of any commercial or financial relationships that could be construed as a potential conflict of interest.

## Publisher’s Note

All claims expressed in this article are solely those of the authors and do not necessarily represent those of their affiliated organizations, or those of the publisher, the editors and the reviewers. Any product that may be evaluated in this article, or claim that may be made by its manufacturer, is not guaranteed or endorsed by the publisher.

## Footnotes

^1^ https://www.hse.gov.uk/seveso/index.htm

^2^ https://usegalaxy.eu

## Supplementary Material

Supplementary Table S1 Gene capture probes used in this study.

## References

[ref1] Amigáň P. Greksák M. Kozánková J. Buezk F. Onderka V. Wolf I. (1990). Methanogenic bacteria as a key factor involved in changes of town gas stored in an underground reservoir. FEMS Microbiol. Ecol. 73, 221–224.

[ref2] Aüllo T. Berlendis S. Lascourrèges J. F. Dessort D. Duclerc D. Saint-Laurent S.. (2016). New bio-indicators for long term natural attenuation of monoaromatic compounds in deep terrestrial aquifers. Front. Microbiol. 7:122. doi: 10.3389/fmicb.2016.00122, PMID: 26904000 PMC4746249

[ref3] Aüllo T. Ranchou-Peyruse A. Ollivier B. Magot M. (2013). *Desulfotomaculum* spp. and related gram-positive sulfate-reducing bacteria in deep subsurface environments. Front. Microbiol. 4:362. doi: 10.3389/fmicb.2013.00362, PMID: 24348471 PMC3844878

[ref4] Badhai J. Ghosh T. S. Das S. K. (2015). Taxonomic and functional characteristics of microbial communities and their correlation with physicochemical properties of four geothermal springs in Odisha, India. Front. Microbiol. 6:1166. doi: 10.3389/fmicb.2015.01166, PMID: 26579081 PMC4620158

[ref5] Baker B. J. Moser D. P. MacGregor B. J. Fishbain S. Wagner M. Fry N. K.. (2003). Related assemblages of sulphate-reducing bacteria associated with ultradeep gold mines of South Africa and deep basalt aquifers of Washington state. Environ. Microbiol. 5, 267–277. doi: 10.1046/j.1462-2920.2003.00408x, PMID: 12662174

[ref6] Basso O. Lascourrèges J. F. Jarry M. Magot M. (2005). The effect of cleaning and disinfecting the sampling well on the microbial communities of deep subsurface water samples. Environ. Microbiol. 7, 13–21. doi: 10.1111/j.1462-2920.2004.00660.x, PMID: 15643931

[ref7] Basso O. Lascourreges J. F. Le Borgne F. Le Goff C. Magot M. (2009). Characterization by culture and molecular analysis of the microbial diversity of a deep subsurface gas storage aquifer. Res. Microbiol. 160, 107–116. doi: 10.1016/j.resmic.2008.10.010, PMID: 19056488

[ref8] Batut B. Gravouil K. Defois C. Hiltemann S. Brugère J. F. Peyretaillade E.. (2018). ASaiM: a galaxy-based framework to analyze microbiota data. Gigascience 7:giy057. doi: 10.1093/gigascience/giy057, PMID: 29790941 PMC6007547

[ref9] Beller H. R. Spormann A. M. Sharma P. K. Cole J. R. Reinhard M. (1996). Isolation and characterization of a novel toluene-degrading, sulfate-reducing bacterium. Appl. Environ. Microbiol. 62, 1188–1196. doi: 10.1128/aem.62.4.1188-1196.1996, PMID: 8919780 PMC167885

[ref10] Berlendis S. Lascourreges J.-F. Schraauwers B. Sivadon P. Magot M. (2010). Anaerobic biodegradation of BTEX by original bacterial communities from an underground gas storage aquifer. Environ. Sci. Technol. 44, 3621–3628. doi: 10.1021/es100123b, PMID: 20380433

[ref11] Callahan B. J. McMurdie P. J. Rosen M. J. Han A. W. Johnson A. J. Holmes S. P. (2016). DADA2: high-resolution sample inference from Illumina amplicon data. Nat. Methods 13, 581–583. doi: 10.1038/nmeth.3869, PMID: 27214047 PMC4927377

[ref12] Casar C. P. Kruger B. R. Osburn M. R. (2021). Rock-hosted subsurface biofilms: mineral selectivity drives hotspots for intraterrestrial life. Front. Microbiol. 12:658988. doi: 10.3389/fmicb.2021.658988, PMID: 33897673 PMC8062869

[ref13] Chapelle F. H. Lovley D. R. (1990). Rates of microbial metabolism in deep coastal plain aquifers. Appl. Environ. Microbiol. 56, 1865–1874. doi: 10.1128/aem.56.6.1865-1874.1990, PMID: 16348227 PMC184524

[ref14] Cornot-Gandolphe S. (2017). Underground gas storage in the world – 2017 Status. Cedigaz Insights No. 22.

[ref16] Daumas S. Magot M. Crolet J. L. (1993). Measurement of the net production of acidity by a sulphate-reducing bacterium: experimental checking of theoretical models of microbially influenced corrosion. Res. Microbial. 144, 327–332. doi: 10.1016/0923-2508(93)90017-v 8248626

[ref17] De Silva G. P. D. Ranjith P. G. Perera M. S. A. (2015). Geochemical aspects of CO_2_ sequestration in deep saline aquifers: A review. Fuel 155, 128–143. doi: 10.1016/j.fuel.2015.03.045

[ref18] DeWeerd K. A. Concannon F. Suflita J. M. (1991). Relationship between hydrogen consumption, dehalogenation, and the reduction of sulfur oxyanions by *Desulfomonile tiedjei*. Appl. Environ. Microbiol. 57, 1929–1934. doi: 10.1128/aem.57.7.1929-1934.1991, PMID: 1892383 PMC183501

[ref19] Dyksma S. Jansen L. Gallert C. (2020). Syntrophic acetate oxidation replaces acetoclastic methanogenesis during thermophilic digestion of biowaste. Microbiome 8:105. doi: 10.1186/s40168-020-00862-5, PMID: 32620171 PMC7334858

[ref21] Escudero C. Oggerin M. Amils R. (2018). The deep continental subsurface: The dark biosphere. Int. Microbiol. 21, 3–14. doi: 10.1007/s10123-018-0009-y, PMID: 30810923

[ref22] Flexer V. Baspineiro C. F. Galli C. I. (2018). Lithium recovery from brines: A vital raw material for green energies with a potential environmental impact in its mining and processing. Sci. Total Environ. 639, 1188–1204. doi: 10.1016/j.scitotenv.2018.05.223, PMID: 29929287

[ref23] Follonier S. Panke S. Zinn M. (2012). Pressure to kill or pressure to boost: a review on the various effects and applications of hydrostatic pressure in bacterial biotechnology. Appl. Microbiol. Biotechnol. 93, 1805–1815. doi: 10.1007/s00253-011-3854-6, PMID: 22290643

[ref24] Frank Y. A. Kadnikov V. V. Gavrilov S. N. Banks D. Gerasimchuk A. L. Podosokorskaya O. A.. (2016). Stable and variable parts of microbial community in siberian deep subsurface thermal aquifer system revealed in a long-term monitoring study. Front. Microbiol. 7:2101. doi: 10.3389/fmicb.2016.02101, PMID: 28082967 PMC5187383

[ref25] Funk M. A. Marsh E. N. Drennan C. L. (2015). Substrate-bound structures of benzylsuccinate synthase reveal how toluene is activated in anaerobic hydrocarbon degradation. J. Biol. Chem. 290, 22398–22408. doi: 10.1074/jbc.M115.670737, PMID: 26224635 PMC4566215

[ref26] Galushko A. Kuever J. (2019). “Desulfobaccaceae,” in Bergey’s Manual of Systematics of Archaea and Bacteria. ed. Whitman W. B. Rainey F. A. Kämpfer P. Trujillo M. E. DeVos P. Hedlund B. (John Wiley & Sons.) doi: 10.1002/9781118960608.fbm00324s

[ref27] Gleeson T. Befus K. M. Jasechko S. Luijendijk E. Bayani Cardenas M. (2015). The global volume and distribution of modern groundwater. Nat. Geosci. 9, 161–167. doi: 10.1038/ngeo2590

[ref28] Gründger F. Jiménez N. Thielemann T. Straaten N. Lüders T. Richnow H.-H.. (2015). Microbial methane formation in deep aquifers of a coal-bearing sedimentary basin, Germany. Front. Microbiol. 6:200. doi: 10.3389/fmicb.2015.00200, PMID: 25852663 PMC4367440

[ref29] Gulliver D. Lipus D. Ross D. Bibby K. (2019). Insights into microbial community structure and function from a shallow, simulated CO_2_-leakage aquifer demonstrate microbial selection and adaptation. Environ. Microbiol. Rep. 11, 338–351. doi: 10.1111/1758-2229.12675, PMID: 29984552

[ref31] Hao L. McIlroy S. J. Kirkegaard R. H. Karst S. M. Fernando W. E. Y. Aslan H.. (2018). Novel prosthecate bacteria from the candidate phylum Acetothermia. ISME J. 12, 2225–2237. doi: 10.1038/s41396-018-0187-9, PMID: 29884828 PMC6092417

[ref32] Hausmann B. Pelikan C. Herbold C. W. Köstlbacher S. Albertsen M. Eichorst S. A.. (2018). Peatland Acidobacteria with a dissimilatory sulfur metabolism. ISME J. 12, 1729–1742. doi: 10.1038/s41396-018-0077-1, PMID: 29476143 PMC6018796

[ref33] Haveman S. A. Pedersen K. (2002). Distribution of culturable microorganisms in Fennoscandian shield groundwater. FEMS Microbiol. Ecol. 39, 129–137. doi: 10.1111/j.1574-6941.2002.tb00914.x, PMID: 19709192

[ref34] Heider J. Szaleniec M. Martins B. M. Seyhan D. Buckel W. Golding B. T. (2016). Structure and function of benzylsuccinate synthase and related fumarate-adding glycyl radical enzymes. J. Mol. Microbiol. Biotechnol. 26, 29–44. doi: 10.1159/000441656, PMID: 26959246

[ref35] Herrmann S. Kleinsteuber S. Chatzinotas A. Kuppardt S. Lueders T. Richnow H. H.. (2010). Functional characterization of an anaerobic benzene-degrading enrichment culture by DNA stable isotope probing. Environ. Microbiol. 12, 401–411. doi: 10.1111/j.1462-2920.2009.02077.x, PMID: 19840104

[ref36] Hershey O. S. Kallmeyer J. Wallace A. Barton M. D. Barton H. A. (2018). High microbial diversity despite extremely low biomass in a deep karst aquifer. Front. Microbiol. 9:2823. doi: 10.3389/fmicb.2018.02823, PMID: 30534116 PMC6275181

[ref37] Hoehler T. M. Jørgensen B. B. (2013). Microbial life under extreme energy limitation. Nat. Rev. Microbiol. 11, 83–94. doi: 10.1038/nrmicro2939, PMID: 23321532

[ref38] Holloway J. Dahlgren R. A. (2002). Nitrogen in rock: occurrences and biogeochemical implications. Global Biogeochem. Cy. 16, 65–61. doi: 10.1029/2002GB001862

[ref601] Hu P. Tom L. Singh A. Thomas B. C. Baker B. J. Piceno Y. M.. (2016). Genome-resolved metagenomic analysis reveals roles for candidate phyla and other microbial community members in biogeochemical transformations in oil reservoirs. mBio. 7, e1669–15. doi: 10.1128/mBio.01669-15, PMID: 26787827 PMC4725000

[ref39] Huang X. Madan A. (1999). CAP3: a DNA sequence assembly program. Genome Res. 9, 868–877. doi: 10.1101/gr.9.9.868, PMID: 10508846 PMC310812

[ref40] Jebbar M. Franzetti B. Girard E. Oger P. (2015). Microbial diversity and adaptation to high hydrostatic pressure in deep-sea hydrothermal vents prokaryotes. Extremophiles 19, 721–740. doi: 10.1007/s00792-015-0760-3, PMID: 26101015

[ref41] Jost A. Violette S. Gonçalvès J. Ledoux E. Guyomard Y. Guillocheau F.. (2007). Long-term hydrodynamic response induced by past climatic and geomorphologic forcing: the case of the Paris basin. France. Phys. Chem. Earth 32, 368–378. doi: 10.1016/j.pce.2006.02.053

[ref42] Kadnikov V. V. Frank Y. A. Mardanov A. V. Beletsky A. V. Ivasenko D. A. Pimenov N. V. (2017). Variability of the composition of the microbial community of the deep subsurface thermal aquifer in western Siberia. Microbiology 86, 765–772. doi: 10.1134/S002626171706008X

[ref43] Kadnikov V. V. Mardanov A. V. Beletsky A. V. Banks D. Pimenov N. V. Frank Y. A.. (2018). A metagenomic window into the 2-km-deep terrestrial subsurface aquifer revealed multiple pathways of organic matter decomposition. FEMS Microbiol. Ecol. 94:10 doi: 10.1093/femsec/fiy152, PMID: 30101334

[ref44] Kadnikov V. V. Mardanov A. V. Beletsky A. V. Karnachuk O. V. Ravin N. V. (2020). Microbial life in the deep subsurface aquifer illuminated by metagenomics. Front. Microbiol. 11:572252. doi: 10.3389/fmicb.2020.572252, PMID: 33013807 PMC7509429

[ref45] Karnachuk O. V. Frank Y. A. Lukina A. P. Kadnikov V. V. Beletsky A. V. Mardanov A. V.. (2019). Domestication of previously uncultivated *Candidatus Desulforudis audaxviator* from a deep aquifer in Siberia sheds light on its physiology and evolution. ISME J. 13, 1947–1959. doi: 10.1038/s41396-019-0402-3, PMID: 30899075 PMC6776058

[ref46] Kleinsteuber S. Schleinitz K. M. Breitfeld J. Harms H. Richnow H. H. Vogt C. (2008). Molecular characterization of bacterial communities mineralizing benzene under sulfate-reducing conditions. FEMS Microbiol. Ecol. 66, 143–157. doi: 10.1111/j.1574-6941.2008.00536.x, PMID: 18637040

[ref47] Kleinsteuber S. Schleinitz K. Vogt C. (2012). Key players and team play: anaerobic microbial communities in hydrocarbon-contaminated aquifers. Appl. Microbiol. Biotechnol. 94, 851–873. doi: 10.1007/s00253-012-4025-0, PMID: 22476263

[ref48] Kliushnikova T. M. Chernyshenko D. V. Kasatkina T. P. (1992). Sul'fatvosstanavlivaiushchaia sposobnost’ bakteriĭ roda pseudomonas [The sulfate-reducing capacity of bacteria in the genus *pseudomonas*]. Mikrobiol. Zh. 54, 49–54. 1584088

[ref49] Kopylova E. Noé L. Touzet H. (2012). SortMeRNA: fast and accurate filtering of ribosomal RNAs in metatranscriptomic data. Bioinformatics 28, 3211–3217. doi: 10.1093/bioinformatics/bts611, PMID: 23071270

[ref50] Kriaa K. Serin J.-P. Contamine F. Cézac P. Mercadier J. (2009). 2-Butyne-1,4-diol hydrogenation in supercritical CO_2_: effect of hydrogen concentration. J. Supercrit. Fluids 49, 227–232. doi: 10.1016/j.supflu.2009.01.004

[ref51] Kuever J. Könneke M. Galushko A. Drzyzga O. (2001). Reclassification of *Desulfobacterium phenolicum* as *Desulfobacula phenolica* comb. nov. and description of strain SaxT as *Desulfotignum balticum* gen. Nov., sp. nov. Int. J. Syst. Evol. Microbiol. 51, 171–177. doi: 10.1099/00207713-51-1-171, PMID: 11211256

[ref52] Kumar S. Stecher G. Li M. Knyaz C. Tamura K. (2018). MEGA X: molecular evolutionary genetics analysis across computing platforms. Mol. Biol. Evol. 35, 1547–1549. doi: 10.1093/molbev/msy096, PMID: 29722887 PMC5967553

[ref53] Kunapuli U. Lueders T. Meckenstock R. U. (2007). The use of stable isotope probing to identify key iron-reducing microorganisms involved in anaerobic benzene degradation. ISME J. 1, 643–653. doi: 10.1038/ismej.2007.73, PMID: 18043671

[ref54] Leandro T. Rodriguez N. Rojas P. Sanz J. L. da Costa M. S. Amils R. (2018). Study of methanogenic enrichment cultures of rock cores from the deep subsurface of the Iberian Pyritic Belt. Heliyon 16:e00605. doi: 10.1016/j.heliyon.2018.e00605, PMID: 29862366 PMC5968172

[ref55] Lemieux A. Shkarupin A. Sharp K. (2020). Geologic feasibility of underground hydrogen storage in Canada. Int. J. Hydrog. Energy 45, 32243–32259. doi: 10.1016/j.ijhydene.2020.08.244

[ref57] Liang B. Wang L. Y. Mbadinga S. M. Liu J. F. Yang S. Z. Gu J. D.. (2015). *Anaerolineaceae* and *Methanosaeta* turned to be the dominant microorganisms in alkanes-dependent methanogenic culture after long-term of incubation. AMB Express 5:117. doi: 10.1186/s13568-015-0117-4, PMID: 26080793 PMC4469597

[ref58] Liang B. Wang L. Y. Zhou Z. Mbadinga S. M. Zhou L. Liu J. F.. (2016). High frequency of *Thermodesulfovibrio* spp. and *Anaerolineaceae* in association with *Methanoculleus* spp. in a long-term incubation of *n*-alkanes-degrading methanogenic enrichment culture. Front. Microbiol. 7:1431. doi: 10.3389/fmicb.2016.01431, PMID: 27695441 PMC5025540

[ref59] Liermann L. Barnes A. S. Kalinowski B. E. Zhou X. Brantley S. (2000). Microenvironments of pH in biofilms grown on dissolving silicate surfaces. Chem. Geol. 171, 1–16. doi: 10.1016/S0009-2541(00)00202-3

[ref60] Magnobosco C. Lin L.-H. Dong H. Bomberg M. Ghiorse W. Stan-Lotter H.. (2018). The biomass and biodiversity of the continental subsurface. Nat. Geosci. 11, 707–717. doi: 10.1038/s41561-018-0221-6

[ref61] Meckenstock R. U. Warthmann R. I. Schafer W. (2004). Inhibition of anaerobic microbial o-xylene degradation by toluene in sulfidogenic sediment columns and pure cultures. FEMS Microbiol. Ecol. 47, 381–386. doi: 10.1016/S0168-6496(03)00303-9, PMID: 19712326

[ref62] Morasch B. Schink B. Tebbe C. C. Meckenstock R. U. (2004). Degradation of o-xylene and m-xylene by a novel sulfate reducer belonging to the genus *Desulfotomaculum*. Arch. Microbiol. 181, 407–417. doi: 10.1007/s00203-004-0672-6, PMID: 15127183

[ref63] Mu A. Boreham C. Leong H. X. Haese R. R. Moreau J. W. (2014). Changes in the deep subsurface microbial biosphere resulting from a field-scale CO_2_ geosequestration experiment. Front. Microbiol. 5:209. doi: 10.3389/fmicb.2014.00209, PMID: 24860559 PMC4030138

[ref64] Nunn H. S. (2015). An Assessment of Microbial Communities and Their Potential Activities Associated with Oil Producing Environments. dissertation. Norman, OK: University of Oklahoma.

[ref65] Nyyssönen M. Hultman J. Ahonen L. Kukkonen I. Paulin L. Laine P.. (2014). Taxonomically and functionally diverse microbial communities in deep crystalline rocks of the Fennoscandian shield. ISME J. 8, 126–138. doi: 10.1038/ismej.2013.125, PMID: 23949662 PMC3869007

[ref66] Parisot N. Denonfoux J. Dugat-Bony E. Peyret P. Peyretaillade E. (2012). KASpOD - a web service for highly specific and explorative oligonucleotide design. Bioinformatics 28, 3161–3162. doi: 10.1093/bioinformatics/bts597, PMID: 23047560

[ref67] Parkes R. J. Cragg B. A. Wellsbury P. (2000). Recent studies on bacterial populations and processes in subseafloor sediments: a review. Hydrogeol. J. 8, 11–28. doi: 10.1007/PL00010971

[ref68] Pedersen K. Arlinger J. Eriksson S. Hallbeck A. Hallbeck L. Johansson J. (2008). Numbers, biomass and cultivable diversity of microbial populations relate to depth and borehole-specific conditions in groundwater from depths of 4-450 m in Olkiluoto, Finland. ISME J. 2, 760–775. doi: 10.1038/ismej.2008.43, PMID: 18432279

[ref69] Peng Y. Leung H. C. M. Yiu S. M. Chin F. Y. L. (2012). IDBA-UD: a *de novo* assembler for single-cell and metagenomic sequencing data with highly uneven depth. Bioinformatics 28, 1420–1428. doi: 10.1093/bioinformatics/bts174, PMID: 22495754

[ref70] Penner T. J. Foght J. M. Budwill K. (2010). Microbial diversity of western Canadian subsurface coal beds and methanogenic coal enrichment cultures. Int. J. Coal Geol. 82, 81–93. doi: 10.1016/j.coal.2010.02.002

[ref71] Pfennig N. Widdel F. Trüper H. (1981). “The dissmilatory sulfate reducing bacteria,” in The Prokaryotes. eds. Starr M. P. Stolp H. Trüpper H. G. Balows A. Schlegel H. G. (Berlin: Springer Verlag), 926–940.

[ref72] Puente-Sánchez F. Arce-Rodríguez A. Oggerin M. García-Villadangos M. Moreno-Paz M. Blanco Y. (2018). Viable cyanobacteria in the deep continental subsurface. Proc. Natl. Acad. Sci. U. S. A. 115, 10702–10707. doi: 10.1073/pnas.1808176115, PMID: 30275328 PMC6196553

[ref73] Quast C. Pruesse E. Yilmaz P. Gerken J. Schweer T. Yarza P.. (2013). The SILVA ribosomal RNA gene database project: improved data processing and web-based tools. Nucleic Acids Res. 41, D590–D596. doi: 10.1093/nar/gks1219, PMID: 23193283 PMC3531112

[ref74] Rabus R. Widdel F. (1995). Conversion studies with substrate analogues of toluene in a sulfate-reducing bacterium, strain Tol2. Arch. Microbiol. 164, 448–451. doi: 10.1007/BF02529744, PMID: 8588748

[ref75] Ranchou-Peyruse M. Auguet J. C. Mazière C. Restrepo-Ortiz C. X. Guignard M. Dequidt D.. (2019). Geological gas-storage shapes deep life. Environ. Microbiol. 21, 3953–3964. doi: 10.1111/1462-2920.14745, PMID: 31314939

[ref76] Ranchou-Peyruse M. Gasc C. Guignard M. Aüllo T. Dequidt D. Peyret P.. (2017). The sequence capture by hybridization: a new approach for revealing the potential of mono-aromatic hydrocarbons bioattenuation in a deep oligotrophic aquifer. Microb. Biotechnol. 10, 469–479. doi: 10.1111/1751-7915.12426, PMID: 27766749 PMC5328808

[ref77] Ribière C. Beugnot R. Parisot N. Gasc C. Defois C. Denonfoux J.. (2016). Targeted gene capture by hybridization to illuminate ecosystem functioning. Microb. Environ. Gen. 1399, 167–182. doi: 10.1007/978-1-4939-3369-3_10 26791503

[ref78] Rognes T. Flouri T. Nichols B. Quince C. Mahé F. (2016). VSEARCH: a versatile open source tool for metagenomics. PeerJ 4:e2584. doi: 10.7717/peerj.2584, PMID: 27781170 PMC5075697

[ref79] Saitou N. Nei M. (1987). The neighbor-joining method: A new method for reconstructing phylogenetic trees. Mol. Biol. Evol. 4, 406–425. doi: 10.1093/oxfordjournals.molbev.a040454, PMID: 3447015

[ref80] Schmieder R. Edwards R. (2011). Quality control and preprocessing of metagenomic datasets. Bioinformatics 27, 863–864. doi: 10.1093/bioinformatics/btr026, PMID: 21278185 PMC3051327

[ref81] Schreiber K. Boes N. Eschbach M. Jaensch L. Wehland J. Bjarnsholt T.. (2006). Anaerobic survival of *Pseudomonas aeruginosa* by pyruvate fermentation requires an Usp-type stress protein. J. Bacteriol. 188, 659–668. doi: 10.1128/JB.188.2.659-668.2006, PMID: 16385055 PMC1347276

[ref82] Soares A. Edwards A. An D. Bagnoud A. Bomberg M. Budwill K.. (2019). A global perspective on microbial diversity in the terrestrial deep subsurface bioRxiv [Preprint]. doi: 10.1101/602672 PMC9993121 36748549

[ref83] Spring S. Lapidus A. Schröder M. Gleim D. Sims D. Meincke L.. (2009). Complete genome sequence of *Desulfotomaculum acetoxidans* type strain (5575). Stand. Genomic Sci. 1, 242–253. doi: 10.4056/sigs.39508, PMID: 21304664 PMC3035247

[ref84] Stemple B. Tinker K. Sarkar P. Miller J. Gulliver D. Bibby K. (2021). Biogeochemistry of the Antrim shale natural gas reservoir. ACS Earth Space Chem. 5, 1752–1761. doi: 10.1021/acsearthspacechem.1c00087

[ref85] Stewart R. Peterson W. (1914). The nitric nitrogen content in the country rock. Utah. Agric. Coll. Exp. Stn. Bull. 134, 421–465.

[ref86] Takami H. Noguchi H. Takaki Y. Uchiyama I. Toyoda A. Nishi S.. (2012). A deeply branching thermophilic bacterium with an ancient acetyl-CoA pathway dominates a subsurface ecosystem. PLoS One 7:e30559. doi: 10.1371/journal.pone.0030559, PMID: 22303444 PMC3267732

[ref87] Tamura K. Stecher G. Peterson D. Filipski A. Kumar S. (2013). MEGA6: molecular evolutionary genetics analysis version 6.0. Mol. Biol. Evol. 30, 2725–2729. doi: 10.1093/molbev/mst197, PMID: 24132122 PMC3840312

[ref88] Tan B. Charchuk R. Li C. Nesbø C. Abu Laban N. Foght J. (2014). Draft genome sequence of uncultivated Firmicutes (*Peptococcaceae* SCADC) single cells sorted from methanogenic alkane-degrading cultures. Genome Announc. 2, e00909–e00914. doi: 10.1128/genomeA.00909-14, PMID: 25212628 PMC4161757

[ref89] Tan S. Liu J. Fang Y. Hedlund B. P. Lian Z. H. Huang L. Y.. (2019). Insights into ecological role of a new deltaproteobacterial order *Candidatus Acidulodesulfobacterales* by metagenomics and metatranscriptomics. ISME J. 13, 2044–2057. doi: 10.1038/s41396-019-0415-y, PMID: 30962514 PMC6776010

[ref90] Taubert M. Vogt C. Wubet T. Kleinsteuber S. Tarkka M. T. Harms H.. (2012). Protein-SIP enables time-resolved analysis of the carbon flux in a sulfate-reducing, benzene-degrading microbial consortium. ISME J. 6, 2291–2301. doi: 10.1038/ismej.2012.68, PMID: 22791237 PMC3504967

[ref91] Tischer K. Kleinsteuber S. Schleinitz K. M. Fetzer I. Spott O. Stange F.. (2013). Microbial communities along biogeochemical gradients in a hydrocarbon-contaminated aquifer. Environ. Microbiol. 15, 2603–2615. doi: 10.1111/1462-2920.12168, PMID: 23809669

[ref92] Ulrich A. C. Edwards E. A. (2003). Physiological and molecular characterization of anaerobic benzene-degrading mixed cultures. Environ. Microbiol. 5, 92–102. doi: 10.1046/j.1462-2920.2003.00390.x, PMID: 12558592

[ref93] Vander Wauven C. Piérard A. Kley-Raymann M. Haas D. (1984). *Pseudomonas aeruginosa* mutants affected in anaerobic growth on arginine: evidence for a four-gene cluster encoding the arginine deiminase pathway. J. Bacteriol. 160, 928–934. doi: 10.1128/jb.160.3.928-934.1984, PMID: 6438064 PMC215798

[ref94] Varliero G. Bienhold C. Schmid F. Boetius A. Molari M. (2019). Microbial diversity and connectivity in deep-sea sediments of the South Atlantic polar front. Front. Microbiol. 10:665. doi: 10.3389/fmicb.2019.00665, PMID: 31024475 PMC6465420

[ref95] Voordouw G. (1995). The genus *Desulfovibrio*: the centennial. Appl. Environ. Microbiol. 61, 2813–2819. doi: 10.1128/aem.61.8.2813-2819.1995, PMID: 16535089 PMC1388543

[ref96] Watanabe M. Fukui M. Galushko A. Kuever J. (2020). “Desulfosarcina,” in Bergey’s Manual of Systematics of Archaea and Bacteria. ed. Whitman W. B. Rainey F. A. Kämpfer P. Trujillo M. E. DeVos P. Hedlund B. (John Wiley & Sons). doi: 10.1002/9781118960608.gbm01020.pub2

[ref97] Watanabe M. Higashioka Y. Kojima H. Fukui M. (2017). *Desulfosarcina widdelii* sp. nov. and *Desulfosarcina alkanivorans* sp. nov., hydrocarbon-degrading sulfate-reducing bacteria isolated from marine sediment and emended description of the genus *Desulfosarcina*. Int. J. Syst. Evol. Microbiol. 67, 2994–2997. doi: 10.1099/ijsem.0.002062, PMID: 28820122

[ref98] Watanabe M. Kojima H. Fukui M. (2013). *Desulfotomaculum intricatum* sp. nov., a sulfate reducer isolated from freshwater lake sediment. Int. J. Syst. Evol. Microbiol. 63, 3574–3578. doi: 10.1099/ijs.0.051854-0, PMID: 23584284

[ref99] Watanabe M. Kojima H. Fukui M. (2018). Review of *Desulfotomaculum* species and proposal of the genera *Desulfallas* gen. Nov., *Desulfofundulus* gen. Nov., *Desulfofarcimen* gen. Nov. and *Desulfohalotomaculum* gen. Nov. Int. J. Syst. Evol. Microbiol. 68, 2891–2899. doi: 10.1099/ijsem.0.002915, PMID: 30028279

[ref100] Wöhlbrand L. Jacob J. H. Kube M. Mussmann M. Jarling R. Beck A.. (2012). Complete genome, catabolic sub-proteomes and key-metabolites of *Desulfobacula toluolica* Tol2, a marine, aromatic compound-degrading, sulfate-reducing bacterium. Environ. Microbiol. 15, 1334–1355. doi: 10.1111/j.1462-2920.2012.02885.x 23088741

[ref101] Wu X. Holmfeldt K. Hubalek V. Lundin D. Åström M. Bertilsson S.. (2016). Microbial metagenomes from three aquifers in the Fennoscandian shield terrestrial deep biosphere reveal metabolic partitioning among populations. ISME J. 10, 1192–1203. doi: 10.1038/ismej.2015.185, PMID: 26484735 PMC5029217

[ref102] Wu X. Pedersen K. Edlund J. Eriksson L. Åström M. Andersson A. F.. (2017). Potential for hydrogen-oxidizing chemolithoautotrophic and diazotrophic populations to initiate biofilm formation in oligotrophic, deep terrestrial subsurface waters. Microbiome 5:37. doi: 10.1186/s40168-017-0253-y, PMID: 28335808 PMC5364579

[ref103] Xia Y. Wang Y. Wang Y. Chin F. Y. Zhang T. (2016). Cellular adhesiveness and cellulolytic capacity in *Anaerolineae* revealed by omics-based genome interpretation. Biotechnol. Biofuels 9:111. doi: 10.1186/s13068-016-0524-z, PMID: 27222666 PMC4877987

[ref105] Youssef N. H. Farag I. F. Rudy S. Mulliner A. Walker K. Caldwell F.. (2019). The wood-Ljungdahl pathway as a key component of metabolic versatility in candidate phylum Bipolaricaulota (Acetothermia, OP1). Environ. Microbiol. Rep. 11, 538–547. doi: 10.1111/1758-2229.12753, PMID: 30888727

[ref106] Zaitseva S. V. Lavrentieva E. V. Radnagurueva A. A. Baturina O. A. Kabilov M. R. Barkhutova D. D. (2017). Distribution of acetothermia-dominated microbial communities in alkaline hot springs of baikal rift zone. PeerJ 5:e3492v1. doi: 10.7287/peerj.preprints.3492v128674656

[ref107] Zhang R. Hedrich S. Ostertag-Henning C. Schippers A. (2018). Effect of elevated pressure on ferric iron reduction coupled to sulfur oxidation by biomining microorganisms. Hydrometallurgy 178, 215–223. doi: 10.1016/j.hydromet.2018.05.003

[ref108] Zhang Y. Li X. Bartlett D. H. Xiao X. (2015). Current developments in marine microbiology: high-pressure biotechnology and the genetic engineering of piezophiles. Curr. Opin. Biotechnol. 33, 157–164. doi: 10.1016/j.copbio.2015.02.013, PMID: 25776196

[ref109] Zuckerkandl E. Pauling L. (1965). “Evolutionary divergence and convergence in proteins,” in Evolving Genes and Proteins. eds. Bryson V. Vogel H. J. (New York: Academic Press), 97–166.

