# Supplementary material for: Microbial Diversity Under the Influence of Natural Gas Storage in a Deep Aquifer
Source: Front Microbiol. 2021 Oct 13;12:688929. doi: 10.3389/fmicb.2021.688929 (PMC8549729; doi:10.3389/fmicb.2021.688929)
Supplement: Supplementary Table S1 — Gene capture probes used in this study. [file Table_1.docx]

**Table S1 : Gene capture probes used in this study**

| Probe# | Sequence |
| --- | --- |
| 1 | ATCGCACCAGCGTGTCAAGCGCCAAGGTACTGGAATACAAGGGAAAAACGATCA |
| 2 | ATCGCACCAGCGTGTGCAAGTGCCAGCCCTACTTCGACCCGGCSGACCTCGGYC |
| 3 | ATCGCACCAGCGTGTAACTTYGAAACGGACCCGGCAAGRAAGGAAGAGTTGYTSGCA |
| 4 | ATCGACCCAGCGTGTTCGAAAGGTACSCCAGCGGCTATSCGCACAAGGAAGACA |
| 5 | ATCGCACCAGCGTGTAACTGGGAAGGCTWCGAGGARATGCGSSYBGAYTTCAAR |
| 6 | ATCGCACCAGCGTGTTACCGSGARATYTTCCCCGGYTCCAACGAYCTKTTCATYCCACTG |
| 7 | ATCGCACCAGCGTGTCGTYGCBGCCAAYTCGYTGGTBGCCATCAARAARCTGGT |
| 8 | ATCGCACCAGCGTGTCCGAGCARCCSAAYGGYTGGYAYAAYCCGATYACVACGATCA |
| 9 | ATCGCACCAGCGTGTGCTYTCCGAGCARCCSAAYGGCTGGCAYAACCCVATCACMCA |
| 10 | ATCGCACCAGCGTGTAATATCVGSACCACCGAGCCYTCCATTGTCTTYCGBTGGCCACTG |
| 11 | ATCGCACCAGCGTGTCGGSYCVAAACYARGARDTTAGTCTTYGAATGTATCCGGGCACTG |
| 12 | ATCGCACCAGCGTGTACGGSTATGAYTGGKCNTAYKCSGAYATGCAGTTGGGKCCCA |
| 13 | ATCGCACCAGCGTGTTTCAGCMAGAACAAYAACGGSGCSACNCCSGARGAAGCC |
| 14 | ATCGCACCAGCGTGTGAYTGGGCCAAYGTTCTCTGYATGTCWCCYGGACTTGTGGCA |
| 15 | ATCGCACCAGCGTGTTGTCTCCAGGACCCACAGGAAGACATGGTGGTTTGAGAACCA |
| 16 | ATCGCACCAGCGTGTAARRCMCAGAAGACCAGGTCRGARGGCGGCGGYTCGMTM |
| 17 | ATCGCACCAGCGTGTGARGCNCAYGACTGGGCCAATGTGCTGTGYATGTCBCCB |
| 18 | ATCGCACCAGCGTGTATTTCRAAACYTTTGARGAYTTDTGGGAAGCCTACCGGRMCACTG |
| 19 | ATCGCACCAGCGTGTATGCCBTTTGTYKCMAGYATTGAYGAYGGYTGCATGGARCCA |
| 20 | ATCGCACCAGCGTGTTATCCGAGCAGCCYAATGGCTGGCAYAAYCCMATYACVA |
| 21 | ATCGCACCAGCGTGTCATCAARAARYTGATTTATGAYGASAAAAARTAYACCWKGCA |
| 22 | ATCGCACCAGCGTGTAAAAARCTGATYTWYGAYGARAARAAATACACCATGAAR |
| 23 | ATCGCACCAGCGTGTTCGTCRAGGCGCTGARRGCCAACTGGGAAGGCYACGAGG |
| 24 | ATCGCACCAGCGTGTTGGAYTTYMWSAAYGCBCCCAARTGGGGCAAYGACGACG |
| 25 | ATCGCACCAGCGTGTCGTYGGGCAGGSTGTCGSCCTKTACATGGAAGTCGGCTC |
| 26 | ATCGCACCAGCGTGTGGMTCRCGCACBGGBCCGACSCCBGAYGGCCGYTTCGGC |
| 27 | ATCGCACCAGCCTGTGGVMGMTTCGGCGGCGARGCKGCKGAYGACCGYGGSATT |
| 28 | ATCGCACCAGCGTGTTGCAGAARAACCAGAARGSCAAYCTGCTSAACCAGMGBY |
| 29 | ATCGCACCAGCGTGTATWGTYCGGGTCKCGGGATTCAGCGCYCGCTTTGTGGAY |
| 30 | ATCGCACCAGCGTGTTTATCGCTCGCACYGAGCAGCAGTTCGGCGCYGARGACYTCA |
| 31 | ATCGCACCAGCGTGTCGCCGAGGACTTCGAATTTCTTAATCTCGAATTAAATTGACACTG |
| 32 | ATCGCACCAGCGTGTTCGTNTCCAGYATYGAYGACGGSTGCATGGAACTBGGCCACT |
| 33 | ATCGCACCAGCGTGTCCGAGCAGRCSAAYGGBTGGCAYAACCCCATHACVACMC |
| 34 | ATCGCACCAGCGTGTGCCATCAAGAARCTSATCTAYGAYGABAARAACACTGCGGCT |
